# Molecular Mechanisms of Gemcitabine Resistance in Cholangiocarcinoma

**DOI:** 10.32604/or.2025.069027

**Published:** 2025-11-27

**Authors:** Sonexai Kidoikhammouan, Charupong Saengboonmee, Sopit Wongkham, Wunchana Seubwai

**Affiliations:** 1 Biomedical Sciences Program, Graduate School, Khon Kaen University, Khon Kaen, 40002, Thailand; 2 Department of Biochemistry, Faculty of Medicine, Khon Kaen University, Khon Kaen, 40002, Thailand; 3 Center for Translational Medicine, Faculty of Medicine, Khon Kaen University, Khon Kaen, 40002, Thailand; 4 Department of Forensic Medicine, Faculty of Medicine, Khon Kaen University, Khon Kaen, 40002, Thailand

**Keywords:** Cholangiocarcinoma, gemcitabine, drug resistance, molecular mechanisms, bile duct cancer, chemotherapy

## Abstract

Cholangiocarcinoma (CCA) is an aggressive cancer originating from bile duct epithelium. Surgical resection remains the primary curative treatment for CCA. However, most CCA patients are diagnosed at an advanced stage, which limits the applicability of surgical resection. Gemcitabine is widely used as a first-line chemotherapeutic agent for unresectable CCA. Its efficacy is often compromised by the development of drug resistance, which leads to poor clinical outcomes and low survival rates of CCA patients. At present, the mechanisms underlying gemcitabine resistance in CCA remain unclear. This review aimed to comprehensively summarize the current knowledge on the molecular mechanisms underlying gemcitabine resistance in CCA and highlight emerging therapeutic strategies that may overcome this resistance. Gemcitabine resistance arises through multiple mechanisms, including reduced drug uptake and increased efflux, impaired drug activation, enhanced DNA repair, apoptosis evasion, aberrations in cell-cycle progression, induction of epithelial–mesenchymal transition, metabolic reprogramming, alteration of tumor, and activation of oncogenic pathways contributes to gemcitabine resistance. A deeper understanding of gemcitabine resistance mechanisms highlights the need for combining gemcitabine with pathway-specific inhibitors, which hold promise for overcoming resistance and improving patient outcomes.

## 1 Cholangiocarcinoma: Classification, Epidemiology, and Treatment

Cholangiocarcinoma (CCA), also known as bile duct cancer, is a highly aggressive and fatal cancer. CCA originates from the epithelial cells lining the bile ducts. This cancer is the second most common primary liver cancer after hepatocellular carcinoma [1,2]. The location of the tumor has a significant influence on classification, clinical presentation, and treatment strategies. Tumors arising within the liver are classified as intrahepatic CCA, whereas those occurring outside the liver are classified as extrahepatic CCA [3,4]. The incidence of CCA has been increasing worldwide over recent decades, especially in Asia. The highest incidence of CCA has been reported in Northeast Thailand, where rates have reached up to 32 cases per 100,000 population [5,6]. In most Western countries, the age-standardized mortality rate for CCA is generally between 0.5 and 2.0 per 100,000 population [7].

Surgical resection remains the most potent curative treatment in the early stages of CCA, which offers the best chance for long-term survival [8,9]. Surgical resection is only feasible in a minority of patients with CCA; however, most are diagnosed at an advanced stage when the tumor has already spread beyond the biliary tree [10]. As such, chemotherapy is an alternative option to manage the CCA patients who are unable to meet the resection criteria. Many chemotherapeutic drugs, such as gemcitabine, capecitabine, and 5-fluorouracil, have been widely tested in clinical trials for CCA patients [10–12]. For nearly a decade, gemcitabine has been used for single and combination therapy with other chemotherapeutic drugs to treat CCA patients.

Gemcitabine is frequently used as a standard first-line chemotherapy agent for patients with advanced CCA [11,13,14]. Gemcitabine primarily exerts its anticancer effects by interfering with DNA synthesis, thereby inhibiting the growth of cancer cells. It is a nucleoside analog that acts as a prodrug. After entering cancer cells via nucleoside transporters, gemcitabine is phosphorylated by deoxycytidine kinase (dCK) into its active metabolites, including gemcitabine diphosphate (dFdCDP) and gemcitabine triphosphate (dFdCTP). dFdCDP inhibits ribonucleotide reductase, which is a key enzyme that converts ribonucleotides to deoxyribonucleotides. Inhibition of ribonucleotide reductase action by dFdCDP results in a reduction of the pool of deoxyribonucleotides, which are needed for DNA synthesis. In addition, dFdCTP is incorporated into DNA during replication, resulting in blocks to further DNA elongation and leading to apoptosis [15,16]. The clinical benefit of gemcitabine therapy in advanced CCA remains limited. The median overall survival (OS) for patients with advanced-stage CCA treated with gemcitabine-based chemotherapy remains less than two years (Table 1). The limited efficacy of gemcitabine is largely attributed to the development of chemoresistance, which can arise through various mechanisms, including reduced drug uptake, increased drug efflux, altered drug metabolism, enhanced DNA repair, and the activation of survival signaling pathways. Therefore, understanding the mechanisms underlying gemcitabine resistance is crucial for developing strategies to overcome this resistance and enhance the effectiveness of gemcitabine-based chemotherapy.

**Table 1:** Clinical outcomes of gemcitabine-based treatment regimens in CCA patients

Treatment regimen	ORR	Median OS (months)	References
Gem	7.9%	8.1	Valle et al. (2010) [17]
Gem + Cis or Gem alone	19%	11.7	
Gem + Cis	21%	10.8	Charoentum C. (2007) [18]
Gem + Cis + Nab-paclitaxel	45%	19.2	Shroff RT. (2019) [19]

Note: ORR, Overall response rate; OS, Overall survival; Gem, Gemcitabine; Cis, Cisplatin.

This review article provides a comprehensive overview of the molecular mechanisms underlying gemcitabine resistance in cholangiocarcinoma, including decreased drug uptake, increased drug efflux through ATP-binding cassette (ABC) transporters, impaired drug activation, enhanced DNA repair, evasion of apoptosis, dysregulation of cell-cycle progression, epithelial mesenchymal transition (EMT), metabolic reprogramming, the influence of the tumor microenvironment (TME), and the activation of survival signaling pathways. It also explores current and emerging therapeutic strategies aimed at overcoming gemcitabine resistance to improve treatment outcomes and survival rates for patients with advanced CCA.

## 2 Reduced Drug Uptake and Increased Drug Efflux Promote Gemcitabine Resistance

Gemcitabine resistance may result from a reduction in the intracellular concentration of the drug. This phenomenon may be caused by reduced drug uptake into cancer cells or increased drug efflux out of cancer cells [20]. The alteration in drug transport mechanisms can significantly limit the amount of gemcitabine that reaches its target within the cell, thereby reducing its effectiveness.

Gemcitabine requires nucleoside transporters, such as the human equilibrative nucleoside transporter 1 (hENT1), to enter cells. Several studies have indicated an association between high hENT1 expression and the efficacy of gemcitabine in various cancers. High hENT1 expression correlated with gemcitabine efficacy in patients with advanced leiomyosarcoma and angiosarcoma. A study in patients with advanced angiosarcoma and leiomyosarcoma found that patients with high hENT1 expression had significantly better progression-free survival (PFS) and OS than patients with low hENT1 expression. Leiomyosarcoma patients treated with gemcitabine exhibited PFS of 6.8 months vs. 3.2 months, and OS of 14.9 months vs. 8.5 months in patients with high hENT1 and low hENT1 expression, respectively. In addition, in angiosarcoma patients with gemcitabine, hENT1 overexpression was associated with a significant improvement in OS (20.6 vs. 10.8 months) and PFS (9.3 vs. 4.5 months) [21]. In CCA patients, the expression levels of hENT1 can act as a significant biomarker in predicting the survival outcomes of patients with advanced CCA undergoing gemcitabine-based chemotherapy. Clinical data from 40 patients with unresectable or recurrent CCA who received gemcitabine (1000 mg/m^2^) plus cisplatin (25 mg/m^2^) demonstrated that hENT1 expression was associated with treatment outcomes. CCA patients with high hENT1 expression had a median PFS of 24 weeks and a median OS of 52 weeks, whereas CCA patients with low hENT1 expression had a median PFS of 11 weeks and a median OS of 26 weeks [22]. Similar finds were reported from Hiroshima University Hospital, Japan [23] and the Cliniques universitaires Saint-Luc, Belgium [24].

*In vitro* studies have demonstrated the direct involvement of hENT1 in mediating gemcitabine resistance in CCA cell lines. First, gemcitabine-resistant CCA cell lines exhibit significantly lower levels of hENT1 expression compared to their parental cells or gemcitabine-sensitive CCA cell lines [25,26]. Second, knockdown of hENT1 expression by siRNA significantly increased the proliferation in gemcitabine-treated CCA cell lines compared with control siRNA [22].

ATP-Binding Cassette (ABC) transporters are a large family of membrane proteins involved in the efflux of a wide range of drugs and other molecules out of cells. These transporters play a significant role in the efflux of chemotherapeutic agents, resulting in a reduction of intracellular concentrations of chemotherapeutic drugs and chemotherapeutic resistance of cancer cells [27]. Overexpression of ABC transporters is strongly associated with tumor aggressiveness, progression, and poor patient prognosis in several cancers [28]. The overexpression of ABC transporters also plays a significant role in the multidrug resistance (MDR) and is associated with poor prognosis of CCA patients. In gemcitabine-resistant CCA cell lines, the overexpression of multidrug resistance-associated protein 1 (MRP1 or ABCC1) has been identified as a significant factor contributing to enhanced drug efflux and resistance [25]. A similar finding was found in CCA patients, MRP1 is significantly overexpressed in CCA tissues compared to non-tumor tissues. The overexpression of MRP1 was significantly correlated with shortened OS of CCA patients [29]. In addition, HuCCT1 cells, an intrahepatic CCA (ICC) cell line, expressed MRP1, MRP2, MRP4, MRP5, and MRP6, whereas KMBC cells, an extrahepatic CCA (ECC) cell line, expressed MRP1, MRP3, MRP4, and MRP5. MRP5 and MRP6 expressions were markedly elevated in HuCCT1 cells and MRP5 in KMBC cells after gemcitabine treatment. MRP5 and MRP6 knockdown significantly increased gemcitabine cytotoxicity in HuCCT1 and KMBC, respectively [30]. These findings indicate that the mechanisms underlying gemcitabine resistance differ between ICC and ECC.

## 3 Impaired Drug Activation Enhances Gemcitabine Resistance

Converting inactive gemcitabine into the active form requires multiple phosphorylation processes. The first and rate-limiting step is catalyzed by dCK. After phosphorylation processes, gemcitabine is converted into dFdCDP and dFdCTP. dFdCDP inhibits ribonucleotide reductase, whereas dFdCTP is incorporated into DNA, leading to chain termination and inhibition of DNA synthesis. Through these mechanisms, gemcitabine exerts its cytotoxic effects on cancer cells.

A reduction of dCK activity or expression can decrease gemcitabine activation and lead to gemcitabine resistance in pancreatic cancer [31,32]. There is a strong negative correlation between dCK levels and gemcitabine resistance in various murine tumors and human tumor xenografts [33]. A study on CCA cell lines demonstrated that forced increasing dCK expression through adenoviral transduction significantly enhances gemcitabine sensitivity [34]. In a phase II study of adjuvant chemotherapy using gemcitabine in patients with resected ICC and ECC, intratumoral expression of dCK was associated with longer recurrence-free survival (RFS). Median RFS was 34.95 months in dCK-positive CCA patients compared to 11.41 months in dCK-negative CCA patients [35]. This information indicated that dCK may serve as both a predictive biomarker and a therapeutic target in CCA.

## 4 Enhanced DNA Repair Mechanisms Increase Gemcitabine Resistance

Gemcitabine exerts its anti-tumor activity by incorporating into DNA, disrupting DNA synthesis, and interfering with the DNA replication process, inducing DNA damage, leading to induction of cancer cell death. Therefore, enhanced DNA repair capacity can counteract the DNA damage induced by gemcitabine and promote gemcitabine resistance in cancer cells [36].

Microarray analysis revealed that p53R2, ribonucleotide reductase regulatory TP53 inducible subunit M2B, is overexpressed in gemcitabine-resistant CCA cell lines [37]. Although the role of p53R2 on gemcitabine response in CCA remains unclear, the role of p53R2 in other cancers has been reported. Data from the TCGA revealed the amplification of p53R2 genes in several types of cancer. In addition, breast cancer patients with p53R2 gene amplification are associated with poor clinical outcomes [38].

## 5 Apoptosis Evasion Facilitates Gemcitabine Resistance

Induction of apoptosis is a common antitumor pathway of several chemotherapeutic drugs. Apoptosis is triggered by intracellular damage signals, especially DNA damage or nucleotide depletion. Cancer cells that acquire the ability to evade or delay apoptosis can survive chemotherapeutic drugs and develop drug resistance [39,40]. The downregulation of BAX and BAK (pro-apoptotic proteins) and caspase enzymes (caspase-3 and caspase-9) plays a significant role in chemotherapeutic resistance. In contrast, the overexpression of Bcl-2 (anti-apoptotic protein) is associated with resistance to chemotherapy in cancer cells [41,42].

The overexpression of anti-apoptotic proteins and the downregulation of pro-apoptotic proteins were commonly reported in CCA patients. Bcl-2 and XIAP expression are significantly higher in CCA tissues compared to non-cancerous tissues. High expression of these anti-apoptosis proteins in CCA is associated with lower OS and higher recurrence rates [43,44]. In contrast, BAX exhibits low expression in CCA when compared to adjacent non-tumor tissues [43]. Not only clinical observations, but also experimental evidence have demonstrated that apoptosis-related proteins play a critical role in gemcitabine resistance in CCA. In gemcitabine-resistant CCA organoids, inhibition of Bcl-xl, an anti-apoptotic protein, could overcome gemcitabine resistance and induce apoptosis [45].

p53 is a well-known tumor suppressor protein that plays a critical role in the cell cycle, DNA repair, senescence, and apoptosis. Under normal conditions, wild-type p53 is expressed at low levels. In contrast, p53 is activated in response to cellular stress induced by DNA damage, leading to cell cycle arrest or apoptosis. In gemcitabine-resistant CCA cell lines, alterations in apoptosis-related proteins and p53 significantly contribute to the development of drug resistance [25]. Elevation of p53 protein level was reported in 58.8%–77% of CCA patients [46–48]. p53 protein expression was significantly associated with poor differentiation and invasion of CCA [48]. Overexpression of the p53 protein often results from mutations, leading to a loss of its tumor suppressor function and/or the acquisition of oncogenic activities. Accumulation of the p53 mutant contributes to apoptosis evasion, chemoresistance. p53 gene mutations were detected in 61.1% CCA patients [48]. Mutant p53 was positively correlated with high hENT1 expression in ICC tissues. ICC patients with >4% mutant p53–positive tumor cells showed significantly higher hENT1 membrane positivity compared to those with ≤4%. In addition, ICC patients treated with adjuvant gemcitabine, those with >4% mutant p53–positive cells had longer DFS compared with ICC patients with ≤4% mutant p53 (18.5 vs. 6 months) [49]. This finding suggests that dysregulation of apoptosis-related proteins plays a critical role in CCA progression and resistance to chemotherapeutic drugs.

## 6 Aberration of Cell-Cycle Progression Contributes to Gemcitabine Resistance

Alterations of cell-cycle regulation are a hallmark of tumor progression and significantly contribute to therapeutic resistance [50]. Gemcitabine primarily targets rapidly dividing cells by incorporating into DNA during the Sphase, leading to chain termination and DNA damage. Therefore, the cytotoxic effect of gemcitabine depends on active cell proliferation and efficient engagement of the cell cycle.

Gemcitabine-resistant CCA cell lines tend to grow more slowly, with longer doubling times compared to their parental cells [25,51]. This slow proliferation rate may help cells evade gemcitabine cytotoxicity. Gemcitabine-resistant cell lines showed increased G1 phase arrest, which may reflect a slower proliferation rate. Cell cycle arrest in cancer cells may also be a protective mechanism against gemcitabine-induced cytotoxicity [52]. A transcriptomic analysis of gemcitabine-resistant CCA cells reveals significant alterations in genes involved in cell-cycle regulation, particularly at the G1/S transition phase, such as MCMs, histone-related genes, Rad51, PCNA, POLA2, DNA ligase I, and E2F1/2 [53].

## 7 EMT Promotes Gemcitabine Resistance

EMT is a dynamic process in which epithelial cells lose their polarity and cell-cell adhesion properties and transform into mesenchymal cells. This phenotypic shift is orchestrated by a network of signaling pathways, including TGF-β, Wnt/β-catenin, and PI3K/Akt, which activate transcription factors that regulate EMT-associated genes. EMT not only drives tumor progression and metastasis in CCA, but also significantly contributes to gemcitabine resistance [54,55].

In pancreatic cancer, EMT involves a shift from E-cadherin to N-cadherin expression. This cadherin switching leads to reduced expression and membrane localization of the equilibrative nucleoside transporter 1 (ENT1). ENT1 is crucial for gemcitabine uptake. Downregulation of ENT1 or hENT1 results in the induction of gemcitabine resistance in CCA [22–24] and pancreatic cancer [56].

## 8 Role of Cancer-Associated Fibroblasts (CAFs) in Gemcitabine Resistance

CAFs are key stromal components of the tumor microenvironment (TME) in CCA. CAFs play a central role in promoting tumor progression and chemoresistance. Derived from normal fibroblasts, CAFs acquire an activated phenotype characterized by enhanced secretion of growth factors (TGF-β, PDGF, FGF), cytokines, and extracellular matrix components. CAFs contribute to chemoresistance through multiple mechanisms, including promoting angiogenesis, remodeling the extracellular matrix, modulating immune responses, and facilitating cancer-cell survival and proliferation [57]. Among these mechanisms, the IL-6/STAT3 signaling pathway has emerged as a key contributor to gemcitabine resistance in CCA. CAF-derived IL-6 activates STAT3 in CCA cells, enhances survival, and reduces drug sensitivity. High serum levels of IL-6 were observed in CCA patients [58]. High IL-6R expression in patient CCA tissues correlates with poor OS and gemcitabine resistance [59].

Targeting CAFs presents a promising strategy to overcome gemcitabine resistance in CCA. Suppressing CAF activation by nintedanib inhibits the secretion of CAF-derived cytokines, such as IL-6 and IL-8, and enhances the antitumor activity of gemcitabine in ICC *in vitro* and *in vivo* [60]. Inhibiting CAF-cancer cell crosstalk using tocilizumab, a monoclonal antibody against the IL-6 receptor, can suppress STAT3 activation and restore gemcitabine sensitivity [59]. In addition, miR-206, a tumor-suppressive microRNA, has been shown to inhibit the transformation of normal fibroblasts into CAFs and enhance gemcitabine efficacy [61]. Together, these findings highlight the therapeutic potential of targeting the CAF-mediated microenvironment to improve treatment outcomes in CCA.

## 9 The Involvement of microRNAs in Regulating Gemcitabine Response

microRNAs (miRNAs) are small non-coding RNAs, approximately 18–25 nucleotides in length. miRNAs regulate gene expression post-transcriptionally by binding to the 3^′^ untranslated region of target mRNAs, leading to either mRNA degradation or translational repression. In cancer, miRNAs play critical roles as oncogenes or tumor suppressors. Aberrant expression of specific miRNAs has been identified in various tumor types and is often associated with tumor initiation, progression, and patient prognosis [62,63].

Several miRNAs have been identified as regulators of gemcitabine resistance in CCA. Oncogenic miRNAs promote gemcitabine resistance, whereas tumor suppressor miRNAs enhance gemcitabine sensitivity [64]. Upregulation of miR-130a-3p was found in gemcitabine-resistant CCA cells. miR-130a-3p promotes gemcitabine resistance by targeting peroxisome proliferator-activated receptor gamma (PPARγ), which has tumor-suppressive functions, including cell-cycle arrest and DNA repair. Inhibition of miR-130a-3p or activation of PPARγ by pioglitazone alleviates resistance and improves gemcitabine efficacy [65].

miR-122, miR-192, miR-29b and miR-155 were significantly increased in serum of CCA patients [66]. miR-29b, miR-205, and miR-221 have been identified as key miRNAs that influence the sensitivity of the HuH28 CCA cell line to gemcitabine. Based on a computational analysis, PIK3R1 and MMP-2 were identified as potential gene targets for these miRNAs. PIK3R1 and MMP-2 are involved in pathways that promote cell survival and proliferation, making them potential targets to enhance chemotherapy effectiveness [67].

## 10 Metabolic Reprogramming of Glucose Metabolism Enhances Gemcitabine Resistance

The relationship between glucose metabolism and gemcitabine resistance in CCA is a complex interplay of metabolic reprogramming and cellular adaptation mechanisms. CCA cells exhibit a high dependency on glucose metabolism, which is often linked to the development of resistance to gemcitabine. Gemcitabine resistance based on glucose metabolism is mediated through various pathways, including alterations in glycolysis, reactive oxygen species (ROS), and cancer stem cell (CSC) phenotypes [68,69].

CCA cells exhibit a preference for glycolysis over oxidative phosphorylation (OXPHOS) for energy production in the presence of oxygen [70,71]. This phenomenon is known as the Warburg effect. This metabolic reprogramming supports rapid cell proliferation and survival under stress conditions. Overexpression of glucose transporter 1 (GLUT1) [72,73], and several key glycolytic-related enzymes, such as pyruvate kinase M2(PKM2) [74], and lactate dehydrogenase A (LDHA) [75,76], have been reported in CCA. Overexpression of these glycolytic-related enzymes correlated with poor prognosis and gemcitabine resistance in CCA.

Overexpression of GLUT1 was significantly associated with non-papillary type, large tumor size, and short survival of CCA patients [72]. Expression of GLUT1 was increased in gemcitabine-treated CCA cell line. Silencing GLUT1 significantly increases the gemcitabine sensitivity of CCA cell *in vitro* and *in vivo* [73].

PKM2 contributes to gemcitabine resistance through both metabolic and non-metabolic mechanisms. Silencing PKM2 expression enhances pancreatic cancer cells to gemcitabine treatment. This effect of PKM2 is associated with the activation of the p38 MAPK pathway and subsequent phosphorylation of p53 at serine 46, culminating in caspase-3/7 activation and PARP cleavage [77]. PKM2 is not only involved in glycolysis but also in regulating oncogenic pathways. Nuclear PKM2 acts as a protein kinase and promotes tumor growth via the activation of oncogenic pathways such as STAT3 [78,79]. Inhibition of PKM2 using siRNA or an inhibitor enhances the gemcitabine sensitivity of ICC *in vitro* and *in vivo* [80].

Although the relationship between LDHA and gemcitabine resistance in CCA has not been directly reported, evidence from other cancers suggests that LDHA plays a critical role in gemcitabine resistance. Novel LDH-A inhibitors exhibit a synergistic effect on gemcitabine-treated pancreatic cancer cells under hypoxic conditions [81]. Altogether, the evidence mentioned above suggests that targeting glycolytic-associated molecules could be a potential strategy to overcome gemcitabine resistance in CCA cells.

ROS play a critical role in various cellular processes, including signal transduction and the regulation of apoptosis. Dysregulated ROS production has been implicated in the development of chemoresistance in cancer cells [82]. High-glucose conditions enhance ROS production in CCA, which plays a pivotal role in promoting the proliferation and migration of CCA cell lines [83].

Gemcitabine-resistant CCA cells shift their metabolism toward glycolysis, which reduces reliance on mitochondrial oxidative phosphorylation. This metabolic shift correlates with CSC-like features, which are associated with chemoresistance. The tumor tissues from CCA patients who do not respond to gemcitabine have low TCA cycle activity and high glucose levels, which is consistent with CSC metabolic signatures [84].

## 11 The Role of Hypoxia in Gemcitabine Resistance

Hypoxia is a common phenomenon in the TME. Rapidly growing tumors often exceed their blood supply, leading to regions of low oxygen tension. This hypoxic microenvironment can significantly influence cancer-cell behavior and treatment response to chemotherapeutic drugs. Hypoxic conditions can promote cancer-cell survival, proliferation, and metastasis, while also reducing the effectiveness of chemotherapeutic agents. Hypoxia is associated with treatment resistance and poor prognosis in CCA [85,86].

Hypoxia-inducible factor 1-alpha (HIF-1α) is a key transcription factor that regulates cellular adaptation to hypoxia and promotes tumor survival, angiogenesis, and metastatic progression. In CCA, overexpression of HIF-1α correlates with advanced tumor stage, increased tumor size, vascular invasion, and intrahepatic metastasis. Patients with HIF-1α-positive tumors exhibit significantly poorer survival outcomes compared to those with HIF-1α-negative tumors [86,87]. HIF-1α also plays a critical role in promoting gemcitabine resistance in CCA through several pathways. Elevated miR-210 sustains HIF-1α activity by suppressing HIF-3α, a negative regulator of HIF-1α. This feed-forward loop promotes cell-cycle arrest at G2/M phase and reduces gemcitabine sensitivity in CCA cells under hypoxia [88]. Overexpression of spindle and kinetochore-associated complex subunit 3 (SKA3) under hypoxia promotes gemcitabine resistance by enhancing fatty acid synthesis via the PARP1/HIF-1α axis. SKA3-overexpressing CCA cells show increased survival and reduced DNA damage after gemcitabine treatment under hypoxic conditions, both *in vitro* and *in vivo* [89].

## 12 Aberrant Activation of Signaling Pathways Mediates Gemcitabine Resistance

### 12.1 EGFR Signaling

The epidermal growth factor receptor (EGFR) is a receptor tyrosine kinase that plays a role in cell proliferation, survival, and differentiation. EGFR is activated by binding to EGF, which triggers a cascade of intracellular signaling events that promote cell growth and survival. EGFR is overexpressed in several human tumors, including lung, breast, and liver cancers. Overexpression of EGFR is associated with aggressive tumor behavior and poor prognosis in several cancers and CCA [90,91]. In ECC, EGFR expression was significantly associated with lymph node metastasis, lymphatic vessels invasion, and perineural invasion. In addition, positive-EGFR expression was also correlated with shorter survival of ICC and ECC patients [91]. Dysregulation of EGFR signaling contributes to gemcitabine resistance. Gemcitabine-induced DNA damage leads to elevated ROS production, which contributes to the activation of stress-related kinases such as Src. This activation suppresses phosphatases that normally dephosphorylate EGFR, resulting in the promotion of phosphorylation of EGFR at tyrosine residues Y845 and Y1173. The activation of EGFR signaling pathways promotes cell survival and proliferation, which in turn reduces gemcitabine efficacy [92,93]. Increased phosphorylation of EGFR is observed in gemcitabine-resistant CCA cells. Erlotinib, a specific inhibitor of EGFR, significantly enhances the antitumor activity of gemcitabine with a synergistic effect in gemcitabine-resistant CCA cells. The synergistic effect of erlotinib on gemcitabine sensitivity suggests a significant role of EGFR signaling in gemcitabine resistance in CCA cells [52].

### 12.2 PI3K/Akt/mTOR Signaling

The PI3K/Akt/mTOR pathway is involved in cell growth, survival, metabolism, and chemotherapeutic resistance. This signaling pathway is activated through stimulation by various growth factors, cytokines, and hormones. Aberrant activation of PI3K/Akt/mTOR pathway has been frequently observed and associated with the progression and poor prognosis of CCA [94,95]. Inhibitions of PI3K/Akt/mTOR pathway by cannabidiol, NVP-BEZ235, and Tanshinone IIA have been shown to reduce proliferation and migration while increasing autophagy, apoptosis, and senescence in CCA cells [94,96,97].

Activation of the PI3K/Akt pathway has been linked to gemcitabine resistance in CCA. This activation supports cancer-cell survival and proliferation, thus diminishing the responsiveness of cancer cells to gemcitabine [98,99]. In contrast, inhibition of the PI3K/Akt pathway has been shown to restore gemcitabine sensitivity [100]. This evidence suggests that targeting the PI3K/Akt/mTOR pathway is a promising therapeutic target to overcome gemcitabine resistance in CCA.

### 12.3 STAT3 Signaling

The signal transducer and activator of transcription 3 (STAT3), a transcription factor which activated by various cytokines and growth factors. Once activated, STAT3 translocated to the nucleus, binds to DNA, and regulates the expression of genes involved in cancer progression [101]. Overexpression of STAT3 was associated with several poor clinical outcomes of CCA patients, including tumor size, vascular invasion, lymph node metastasis, shorter OS, and DFS [59,102]. IL-6 is a cytokine that activates the STAT3 signaling pathway. Increased IL-6/STAT3 activation can promote cancer-cell survival and reduce the sensitivity to gemcitabine [59]. Blockade of IL-6R can inhibit the CAF-CCA interaction and enhance gemcitabine sensitivity [59]. This approach represents a promising strategy for overcoming chemoresistance in CCA.

### 12.4 Wnt**/β**-Catenin Pathway

Wnt/β-catenin plays a crucial role in regulating various cellular processes in cancer cells, such as proliferation, differentiation, migration, apoptosis, and chemoresistance. Inhibition of Wnt/β-catenin signaling pathway using inhibitors (C59, XAV939, and PRI724) caused a reduction in the expression of its downstream target genes, namely AXIN2 and survivin. Furthermore, Supplementation of C59, XAV939, and PRI724 significantly enhanced the anti-tumor activity of gemcitabine in ICC and ECC cell lines [103].

## 13 Novel Therapeutic Strategies to Overcome Gemcitabine Resistance

**Figure 1:**
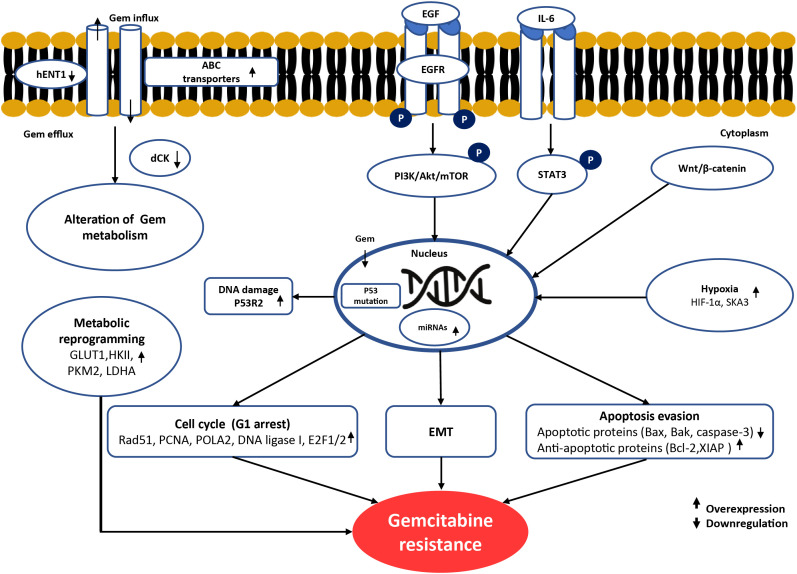
Schematic summary of the molecular mechanisms underlying gemcitabine resistance in cholangiocarcinoma. Gemcitabine resistance involves multiple alterations. At the cell membrane, reduced drug uptake through downregulation of hENT1 and increased drug efflux via ABC transporters limit intracellular gemcitabine accumulation. In cancer cells, impaired gemcitabine activation occurs due to reduced dCK expression. Dysregulation of apoptosis is observed through downregulation of BAX, BAK, and caspase-3, together with overexpression of anti-apoptotic proteins such as Bcl-2 and XIAP. Cell-cycle arrest at G1 phase driven by alterations in Rad51, PCNA, POLA2, DNA ligase I, and E2F1/2 reduces gemcitabine cytotoxicity by limiting incorporation during the S phase. Metabolic reprogramming, including overexpression of GLUT1, HKII, PKM2, and LDHA, enhances glycolysis and promotes gemcitabine resistance. EMT and aberrant activation of oncogenic signaling pathways, such as EGFR, PI3K/Akt/mTOR, STAT3, and Wnt/β-catenin, support survival and proliferation under stress conditions induced by gemcitabine. In the nucleus, enhanced DNA repair mediated by p53R2 (RRM2B) counteracts gemcitabine-induced DNA damage. In the tumor microenvironment, hypoxia-related factors (HIF-1α and SKA3) can induce gemcitabine resistance by promoting metabolic and signaling adaptations. Upward arrows (↑) indicate overexpression/activation, whereas downward arrows (↓) indicate downregulation/inactivation of the corresponding molecules. Created using Microsoft PowerPoint. Kidoikhammouan, S. (2025)

The mechanistic insights into gemcitabine resistance suggest potential therapeutic strategies for restoring drug sensitivity and improving clinical outcomes. The potential molecules related to gemcitabine resistance in CCA are summarized and demonstrated in Fig. 1 and Table 2.

**Table 2:** Key molecules associated with gemcitabine resistance in CCA and clinical relevance

Key pathway/molecules	*In vitro/In vivo*	Clinical relevance	Ref.
**Drug uptake and drug efflux**			
hENT1	√	High hENT1 expression was associated with longer survival in CCA patients receiving gemcitabine-based therapy.	[22–24,26]
MRP1, MRP5, MRP6	√	Overexpression of MRP1 was correlated with shorter OS of CCA patients.	[29,30]
**Drug activation**			
dCK	√	High dCK expression was associated with significantly longer RFS in resected CCA patients treated with adjuvant gemcitabine.	[34,35]
**DNA repairing**			
p53R2	√	NA	[37]
**Apoptosis**			
Bcl-2	√	In ICC patients, high Bcl-2 expression is associated with poor OS and higher recurrence.	[25,43,45]
XIAP	√	Overexpression of XIAP was associated with poorer OS in CCA patients.	[44]
BAX	√	In ICC patients, low BAX expression is associated with poor OS and higher recurrence.	[25,43]
p53	√	Overexpression of p53 was observed in CCA patients. ICC patients with mutant p53 > 4% of tumor cells had significantly longer DFS when treated with adjuvant gemcitabine.	[46,47,49]
**Cancer-associated fibroblasts**			
IL-6	√	High serum level of IL-6 was detected in CCA patients	[58–60]
**microRNAs**			
miR-206	√	Downregulation of miR-206 was associated with shorter OS and DFS of ICC patients.	[61]
miR 130a-3p	√	High miR-130a-3 was significantly associated with lower DFS/OS and higher vascular invasion in ICC patients.	[65]
miR 29b, miR 205, and miR 221	√	Serum miR-29b was significantly increased in CCA patients.	[66,67]
**Glucose metabolism**			
GLUT1	√	GLUT1 overexpression was associated with shorter OS and DFS of ICC patients.	[72,73]
PKM2	√	PKM2 was overexpressed and correlated with the survival of CCA patients.	[74,80,104]
**Hypoxia**			
HIF-1α	√	HIF-1α expression was significantly correlated with higher stage, intrahepatic metastasis, shorter OS, and DFS of ICC patients.	[86,87,89]
**Signaling Pathways**			
EGFR	√	EGFR overexpression was associated with lymph node metastasis, tumor stage, lymphatic vessel invasion, and perineural invasion in ECC. EGFR expression was a significant poor prognostic factor and recurrence in ICC.	[52,91]
PI3K/Akt/mTOR	√	Overexpression of p-AKT and p-mTOR was found in ECC patients. In addition, overexpression of p-AKT1 and p-MTOR was associated with better survival in ICC patients.	[100,105,106]
STAT3	√	Overexpression of STAT3 was associated with several poor clinical outcomes of CCA patients, including tumor size, vascular invasion, lymph node metastasis, shorter OS, and DFS.	[59,102]
Wnt/beta-catenin	√	Beta-catenin was expressed in 38.6% of ICC patients.	[103,107]

Note: ECC, Extrahepatic cholangiocarcinoma; ICC, Intrahepatic cholangiocarcinoma; OS, Overall survival; DFS, Disease-free survival; NA, Not applicable.

Several chemotherapeutic and chemosensitizing agents targeting gemcitabine resistance pathways have been evaluated in preclinical models and clinical trials of CCA and other cancers, as summarized in Table 3.

**Table 3:** Potential chemotherapeutic or chemosensitizer agents against molecular targets associated with gemcitabine resistance in CCA

Target/Drugs	Clinical trial details (Treatment regimen)	Ref.
BCL-2/Navitoclax	Phase I: Solid tumors (Combination with gemcitabine)	[111]
STAT3**/**Napabucasin	Phase III: Pancreatic cancer (Combination with gemcitabine and nab-paclitaxel)	[116]
IL6/Tocilizumab	Phase II: Pancreatic cancer (Combination with gemcitabine and nab-paclitaxel)	[114]
EGFR/Erlotinib	Phase III: CCA (Combination with gemcitabine and oxaliplatin)	[113]
p53**/**p53MVA	Phase I: Ovarian cancer (Combination with gemcitabine)	[112]
Wnt**/**Ipafricept	Phase Ib: Pancreatic cancer (Combination with gemcitabine and nab-paclitaxel)	[113]

One of the promising strategies involves targeting drug transport mechanisms. For example, enhancing the expression or function of hENT1, which mediates gemcitabine uptake. On the other hand, inhibition of ABC transporters could prevent drug efflux and increase intracellular gemcitabine concentration in cancer cells. VX-710 (biricodar, INCEL), an inhibitor of P-glycoprotein (P-gp) and MRP1, has been investigated as a chemosensitizer in several cancers in preclinical and clinical studies. Although the combination of VX-710 with gemcitabine has not yet been investigated, studies with other chemotherapeutic agents have demonstrated promising results, such as recurrent small-cell lung cancer [108], ovarian cancer [109]. Similarly, dofequidar fumarate (MS-209), an orally bioavailable quinoline compound that inhibits P-gp and MDR-1, has also demonstrated potential in overcoming multidrug resistance in cancer therapy [110].

Another possible approach is the restoration of gemcitabine activation through the upregulation of dCK, the rate-limiting enzyme responsible for gemcitabine phosphorylation. Preclinical studies have demonstrated that increased dCK expression sensitizes CCA cells to gemcitabine, which suggests the potential for gene therapy or pharmacological induction.

Targeting enhanced DNA repair pathways also holds therapeutic potential. Upregulation of ribonucleotide reductase subunit p53R2 has been implicated in resistance by maintaining deoxyribonucleotide pools necessary for DNA repair. Inhibiting such compensatory repair mechanisms may sensitize tumor cells to gemcitabine-induced DNA damage.

Apoptosis evasion is a key contributor to gemcitabine in CCA. Therapies that restore pro-apoptotic signaling, such as upregulating BAX and caspases or inhibiting anti-apoptotic proteins like Bcl-2 and XIAP, have been proposed to resensitize cells to gemcitabine. Nowadays, there are several BCL-2 family inhibitors have been used in clinical trials, such as Venetoclax (a selective small-molecule inhibitor for targeting of Bcl-2) and Navitoclax. Navitoclax or ABT-263 is a pan-BCL-2 family inhibitor that inhibits Bcl-XL, Bcl-2, and Bcl-W. This pan-BCL-2 family inhibitor has been used in phase I and II clinical trials of several cancers. A phase I clinical trial on the combination of navitoclax with gemcitabine in patients with solid tumors found that the combination was generally well tolerated and exhibited a favorable safety profile in patients with advanced solid tumors [111].

Moreover, targeting mutant p53, which contributes to apoptosis resistance and chemoresistance, represents another promising avenue. In the phase 1 clinical trial, the combination of p53-expressing modified vaccinia Ankara virus (p53MVA) with gemcitabine was used to treat ovarian cancer patients. The result demonstrated that in 11 patients who received at least one treatment cycle, no complete responses were observed. Three patients achieved stable disease, and one patient had a partial response (64% tumor reduction) [112].

Alterations in cell-cycle progression contribute to a reduced cytotoxic response to gemcitabine. Agents that modulate cell-cycle regulators and promote S-phase entry may restore drug sensitivity by enhancing DNA incorporation of gemcitabine.

Emerging evidence also highlights the role of EMT and non-coding RNAs in chemoresistance. Inhibiting EMT-associated signaling pathways (such as TGF-β and Wnt/β-catenin) can reverse EMT-mediated drug resistance. The Phase Ib study of ipafricept (OMP-54F28), a Wnt pathway inhibitor, combined with gemcitabine and nab-paclitaxel in untreated stage IV pancreatic cancer patients showed that ipafricept can be administered with standard chemotherapy with reasonable tolerance. The study reported a clinical benefit rate of 81%, including 34.6% partial responses and 46.2% stable disease. Median progression-free survival was 5.9 months, and median overall survival was 9.7 months [113].

The modulation of miRNAs, such as suppressing oncogenic miR-130a-3p or restoring tumor-suppressive miR-424-5p, has demonstrated potential in sensitizing CCA cells to gemcitabine.

The TME, particularly CAFs, contributes to gemcitabine resistance via the secretion of cytokines such as IL-6, which activate the STAT3 signaling pathway in tumor cells. Targeting this interaction using monoclonal antibodies against IL-6R (tocilizumab) or reprogramming CAFs through miRNAs such as miR-206 may provide therapeutic benefit. The randomized Phase II study evaluated the combination of tocilizumab, an anti-IL-6 receptor antibody, with a first-line chemotherapy agent of gemcitabine and nab-paclitaxel in patients with advanced pancreatic cancer. Overall survival at 6 months showed no statistically significant difference between the combination (tocilizumab + gemcitabine/nab-paclitaxel) and chemotherapy alone (68.6% vs. 62.0%). However, overall survival at 18 months improved significantly with tocilizumab (27.1% vs. 7.0%) [114].

Metabolic reprogramming, including enhanced glycolysis and reduced oxidative phosphorylation, has also been linked to chemoresistance. Inhibitors of GLUT1 and key glycolytic enzymes, such as HK2, PKM2, and LDHA, have demonstrated efficacy in reversing resistance and restoring gemcitabine sensitivity.

Furthermore, hypoxia-induced pathways involving HIF-1α and its downstream effectors (miR-210 and SKA3) have been identified as mediators of drug resistance under low oxygen conditions. Therapeutic strategies targeting hypoxia signaling may be effective in overcoming resistance in hypoxic tumor regions.

Finally, aberrant activation of oncogenic signaling pathways, including EGFR, PI3K/Akt/mTOR, and STAT3, plays a central role in gemcitabine resistance. Pharmacological agents that inhibit these pathways, such as erlotinib (EGFR inhibitor), NVP-BEZ235 (PI3K/mTOR dual inhibitor), or cannabidiol (Akt/mTOR modulator), have shown synergistic effects with gemcitabine in preclinical models of CCA. A randomized phase 3 study investigated the efficacy of gemcitabine and oxaliplatin (GEMOX) with or without erlotinib in advanced CCA patients. The study found that more patients had an objective response in the chemotherapy plus erlotinib group than in the chemotherapy alone (40 patients vs. 21 patients) [115].

Collectively, these findings underscore the need for integrated therapeutic strategies that simultaneously target multiple resistance pathways. Further preclinical and clinical investigations are essential to validate these approaches and facilitate their translation into effective treatment regimens for patients with gemcitabine-resistant CCA.

## 14 Summary

In summary, overcoming gemcitabine resistance in CCA requires a multifaceted therapeutic approach that targets the diverse molecular alterations exploited by resistant cancer cells. These mechanisms include reduced drug uptake, enhanced drug efflux via ABC transporters, impaired drug activation, upregulated DNA repair pathways, evasion of apoptosis, dysregulation of cell-cycle progression, activation of EMT, metabolic reprogramming, influences from the TME, and aberrant activation of pro-survival signaling cascades. Targeting these pathways represents a promising strategy to restore gemcitabine sensitivity in CCA cells.

This intricate network of gemcitabine resistance pathways highlights the need for comprehensive therapeutic strategies that target multiple molecules within CCA cells. Rational combination therapies such as combination treatment of gemcitabine with agents that improve drug uptake, induce DNA damage, restore apoptosis, or inhibit key oncogenic pathways hold promise for overcoming chemoresistance.

Moreover, the emergence of computational drug discovery platforms and AI-based predictive models offers a powerful framework to identify compounds capable of reversing resistance-associated gene-expression profiles and predicting synergistic drug combinations. Tools such as the Connectivity Map (CMap), iLINCS, and L1000FWD facilitate transcriptomics-guided drug repurposing by matching disease-specific signatures with compounds that elicit opposing transcriptional effects. In parallel, deep learning models including MatchMaker and DeepSynergy enable synergy prediction based on molecular profiles and drug structure, thereby providing insight into rational combination strategies.

Through the strategic integration of mechanistic understanding, computational drug discovery, and precision-medicine approaches, the therapeutic landscape for gemcitabine-resistant CCA may be transformed from a clinical challenge to a tractable CCA with enhanced therapeutic opportunities and improved patient prognosis. Future research should prioritize validating promising targets in large-scale clinical trials, incorporating multi-omics data into predictive frameworks, and translating these findings into clinically applicable regimens. Such strategies may ultimately improve survival outcomes and quality of life for patients with CCA.

## Data Availability

Not applicable.
